# Simultaneous Improvement of Hole and Electron Injection in Organic Field-effect Transistors by Conjugated Polymer-wrapped Carbon Nanotube Interlayers

**DOI:** 10.1038/srep10407

**Published:** 2015-05-22

**Authors:** Seung-Hoon Lee, Dongyoon Khim, Yong Xu, Juhwan Kim, Won-Tae Park, Dong-Yu Kim, Yong-Young Noh

**Affiliations:** 1 Department of Nanobio Materials and Electronics, School of Material Sciences and Engineering, Heeger Center for Advanced Materials, Gwangju Institute of Science and Technology (GIST), 261 Cheomdan-gwagiro (Oryong-dong), Buk-gu, Gwangju 500-712, Republic of Korea; 2 Department of Energy and Materials Engineering, Dongguk University, 26 Pil-dong, 3-ga, Jung-gu, Seoul 100-715, Republic of Korea

## Abstract

Efficient charge injection is critical for flexible organic electronic devices such as organic light-emitting diodes (OLEDs) and field-effect transistors (OFETs). Here, we investigated conjugated polymer-wrapped semiconducting single-walled carbon nanotubes (s-SWNTs) as solution-processable charge-injection layers in ambipolar organic field-effect transistors with poly(thienylenevinylene-co-phthalimide)s. The interlayers were prepared using poly(9,9-di-n-octylfluorene-alt-benzothiadiazole) (F8BT) or poly(9,9-dioctylfluorene) (PFO) to wrap s-SWNTs. In the contact-limited ambipolar OFETs, the interlayer led to significantly lower contact resistance (*R*_*c*_) and increased mobilities for both holes and electrons. The resulting PTVPhI-Eh OFETs with PFO-wrapped s-SWNT interlayers showed very well-balanced ambipolar transport properties with a hole mobility of 0.5 cm^2^V^-1^S^-1^ and an electron mobility of 0.5 cm^2^V^-1^S^-1^ in linear regime. In addition, the chirality of s-SWNTs and kind of wrapping of conjugated polymers are not critical to improving charge-injection properties. We found that the improvements caused by the interlayer were due to the better charge injection at the metal/organic semiconductor contact interface and the increase in the charge concentration through a detailed examination of charge transport with low-temperature measurements. Finally, we successfully demonstrated complementary ambipolar inverters incorporating the interlayers without excessive patterning.

Charge injection from metal electrodes into organic semiconductors (OSCs) is one of the critical issues in realizing high-performance organic electronic devices, because most of these devices contain Schottky-type metal-semiconductor contacts^1^,^2^. In conventional silicon-based devices, heavily doping in electrodes enables the fabrication of Ohmic contacts, permitting efficient charge injection. However, such an efficient doping technique on an atomic scale is unfeasible for organic devices^3^,^4^,^5^. Therefore, the performance of various organic devices (e.g., organic light-emitting diodes (OLEDs), and organic field-effect transistors (OFETs)) is severely limited by the poor injection properties.^2^,^3^,^6^,^7^ For instance, the large Schottky barriers raise the threshold voltage (V_th_) of OFETs and subsequently the operating voltage and power consumption of the composed integrated circuits (ICs)^2^,^8^. With downscaling for high-density integration and high-speed operation, the contact limitations become more and more serious than in normal-sized devices^2^,^3^,^9^. As the channel length (*L*) of transistors shrinks, the channel transport becomes more “current demanding,” but the charge injection/extraction capability at the metal/semiconductor interfaces does not increase in proportion to *L*. As a result, injection-related limitations start to distort the overall transport characteristics and gradually become a crucial problem for device downscaling. In addition to the large Schottky barrier, inefficient charge injection could also arise from significant charge trapping, structural OSC disorders, and additional effects induced by charge transfer at the contacts in downscaled devices^10^.

A number of approaches have been developed to optimize the charge-injection properties at the metal–OSC interface, most of them related to the modification of interface electrical or chemical properties^11^. The interface-modification techniques can be classified into three types.

The first involves tuning the work function (Φ_WF_) of metal electrodes through the interface dipole by using self-assembled monolayers (SAMs) or other polymer interlayers. The SAMs of thiols and aliphatic polymers with amine groups (e.g., polyethylenimine ethoxylated (PEIE) and branched polyethylenimine (PEI)) as surface modifiers have been widely applied in this respect^12^,^13^,^14^,^15^. However, the SAM approach is limited in its application to specific metal electrodes such as gold and silver, and it is not appropriate for conjugated polymers that necessitate a high annealing temperature of over 200 °C to improve crystallinity. In addition, it is difficult to guarantee high device uniformity over a large area using polymer interlayers containing amine functional groups, because the optimum thickness of the polymeric interlayers is very thin (~2 nm) due to their insulating properties^15^,^16^.

The second method involves doping OSCs selectively at the semiconductor–electrode interface. Chemical doping at the interface increases the conductivity of OSCs locally and effectively reduces charge-trapping effects, as the Fermi level is shifted toward the corresponding transport bands, filling the resident trap states therein. Moreover, with greater charge concentrations, the thickness of the depletion region within the OSC at the metal–OSC interface is significantly decreased. Thus, the more efficient field emission (tunneling) replaces thermionic emission through a large Schottky barrier and starts to contribute predominately to the overall charge injection^17^,^18^. Note that doping increases the “OFF” current and decreases the ON/OFF ratio of OFETs. In addition, the exact doping mechanism, and the concentration and location of the dopant in OSCs are still not well understood. Thus, more comprehensive research is needed to apply these techniques for further optimization of devices.

The last method involves introducing metal-oxide interlayers to the metal–OSC interface. The barrier height of charge injection from the source/drain electrodes to the active OSC layers must be effectively reduced by altering the interface dipoles or weakened by gap state-assisted hopping^19^,^20^,^21^,^22^. However, most metal-oxide-based interlayers require high-vacuum-based deposition processes with high-temperature annealing processes and degrade the ambient stability of devices in particular interlayers for electron injection^20^,^21^.

Until now, conventional interlayers have generally been applied to improve charge injection for only one-polarity charge carriers, either holes or electrons, since the interlayers change the Φ_WF_ of metal electrodes in one direction, either high or low. Interlayers that can enhance both hole and electron injection are very rare, because the underlying mechanisms of these interlayers are rather different from the conventional interlayers that tune Φ_WF_ of metal electrodes by inducing interface dipoles and/or introducing dopants close to the interface. However, a technique that improves the injection of both charge carriers is needed for ambipolar-based sensors^23^, ICs^24^,^25^, and ambipolar light-emitting field-effect transistors (FETs)^8^. Xie *et al.* recently reported enhanced injection of both holes and electrons in organic single-crystal transistors using Au/single-walled carbon nanotube (SWNT) electrodes^10^. The one-dimensional (1D) structure of SWNTs creates a strong electrostatic field that is known in SWNT-based FETs and has been evidenced to facilitate charge tunneling through Schottky contacts without producing large energetic barriers. Therefore, the use of SWNT electrodes for ambipolar charge injection is attractive^26^,^27^. Recently, several research groups reported a method of obtaining highly purified semiconducting SWNT (s-SWNT) dispersion through the wrapping of conjugated polymers^28^,^29^,^30^,^31^,^32^. More recently, using this sorting method, Gwinner *et al.* reported ambipolar light-emitting FETs with enhanced light emission intensity by adding a small amount of s-SWNTs to the semiconducting layer^8^.

In this paper, we report a simple but effective method of improving both hole and electron injection in ambipolar OFETs with poly(thienylenevinylene-co-phthalimide)s (PTVPhI-Eh) (Fig. 1b) using a conjugated polymer-wrapped s-SWNT as solution-processable charge-injection interlayers. The solution-processed interlayers are fabricated by selective wrapping of poly(9,9-di-n-octylfluorene-alt-benzothiadiazole (F8BT) or poly(9,9-dioctylfluorene) (PFO) on the surface of s-SWNT with a specific chirality through π–π interaction. The hole and electron mobilities are significantly enhanced (by approximately 3–100 times) by inserting an F8BT-wrapped s-SWNT interlayer (F8BT:s-SWNT) and PFO-wrapped s-SWNT interlayer (PFO:s-SWNT) through much-reduced contact resistances (*R*_*c*_), leading to well-balanced ambipolar transport. After a detailed investigation of charge transport by low-temperature measurements and with mobility calculations based on a solid transport model, we found that the Au–interlayer–OSC interface provides much better charge injection for both holes and electrons as compared to the pristine Au–OSC interface, highlighting the importance of the interlayers for ambipolar OFET applications.

## Results

**Figure 1:**
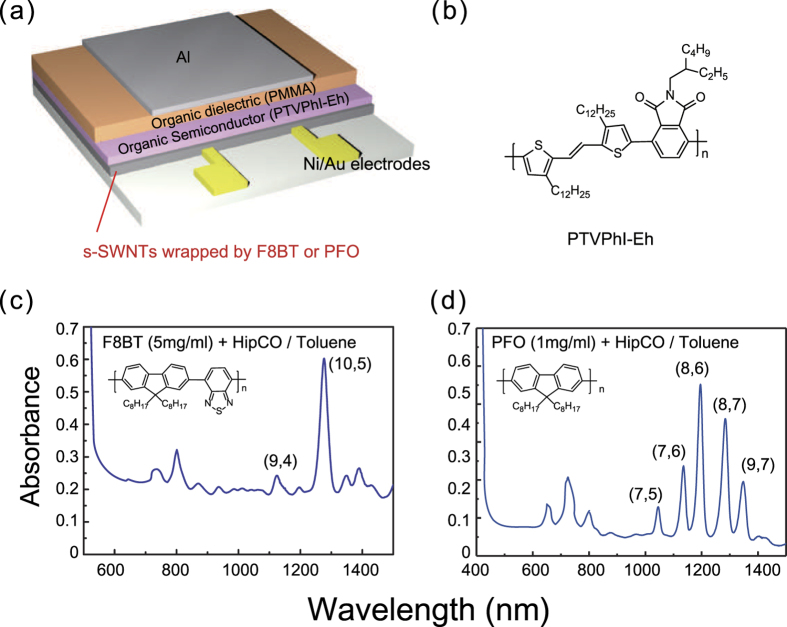
(**a**) Schematic of TGBC OFET device structure, (**b**) Molecular structure of PTVPhI-Eh, (**c**) Optical absorption spectra of as-dispersed F8BT (5 mg/ml)- and (**d**) PFO (1 mg/ml)-wrapped s-SWNTs in toluene solvent after mild centrifugation.

A top-gate and bottom-contact (TGBC) staggered device structure (Fig. 1a) was used, because the interlayer can be applied on the pre-patterned Au source and drain electrodes by a simple solution process and a semiconducting layer can be effectively passivated by the overlying gate dielectric and gate electrode layer to minimize degradation of the device by oxygen and moisture in the air. The interlayer solution was prepared by selectively wrapping s-SWNTs using F8BT or PFO, similar to the previously reported processes involving only simple sonication followed by a mild centrifugation step^33^,^34^. Next, 2 mg of SWNT produced by high-pressure carbon monoxide (HiPCO) method and 25 mg of F8BT or 5 mg of PFO were mixed in 5 ml toluene for the dispersion process. After tip sonication and the mild centrifugation step (10,000 g for 30 min), the interlayer solutions were obtained. These procedures are described in more detail in the method section. This method of s-SWNT sorting with a low-speed centrifugation is easy and efficient, but the application of this technique in the literature is very rare, because the excess polymer is too much to have a negligent effect on device performance^35^. As a result, these s-SWNTs sorted by conjugated polymer wrapping require further processes to remove the excess polymers and obtain high purity s-SWNT (i.e., ultracentrifugation or filtration) for FET applications as active layers^8^,^28^,^29^,^30^,^35^. Note that unlike most studies, we directly apply SWNTs wrapped by F8BT or PFO solution using a mild centrifugation as contact interlayers between source/drain electrodes and OSC layers. Fig. 1(c) and (d) show the absorption spectra of F8BT- and PFO-wrapped SWNTs dispersed in toluene obtained using this approach. As previously reported, F8BT selectively wraps (9,4) and (10,5) s-SWNTs, and PFO is selective for five different s-SWNT species corresponding to (7,5), (7,6), (8,6), (8,7), and (9,7)^33^,^34^. In order to determine the concentrations of s-SWNTs in PFO:s-SWNT or F8BT:s-SWNT solution, we used the absorption cross-section (*c.a.* 1.7 × 10^−17^ cm^2^) for the E^11^ absorption peak of s-SWNT^36^. The concentration of s-SWNTs was approximately 1.2 μg/ml for both F8BT:s-SWNT and PFO:s-SWNT solution, because the optical densities of the two interlayer solutions were similar ((10,5) s-SWNT in F8BT:s-SWNT and (8,6) in PFO:s-SWNT ≈ 0.6). On the other hand, we intentionally applied different concentrations of F8BT (5 mg/ml) and PFO (1 mg/ml) to obtain the similar concentrations of s-SWNTs in the interlayer solutions, since the two polymers showed different wrapping efficiencies.

**Figure 2:**
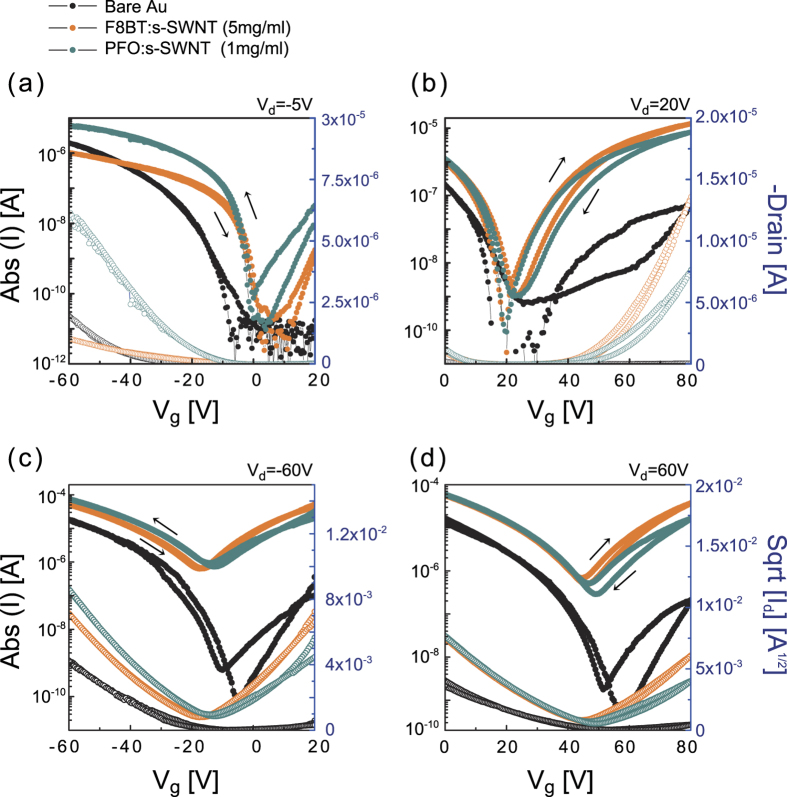
*P*-channel and *n*-channel transfer characteristics in linear (**a,b**) and saturation (**c,d**) regimes of ambipolar PTVPhI-Eh OFETs with s-SWNTs wrapped by F8BT or PFO as a charge-injection interlayer (*L* = 20 μm, *W*/*L* = 50, *C*_i_ = 6.2 nFCm^−2^).

As shown in Fig. 2, the transfer curves of PTVPhI-Eh OFETs with PFO:s-SWNT and F8BT:s-SWNT interlayers exhibited remarkably improved *p*- and *n*-channel characteristics in OFETs. The incorporation of the interlayers effectively improved all basic parameters of transistors, including both hole and electron mobility (*μ*_h_ and *μ*_e_), ON current and *V*_th_, and small subthreshold swing (*SS*). Details are summarized in Table 1. In the linear regime (*V*_ds_ = −5 V) and saturation regime (*V*_ds _= −60 V), the average *μ*_h_ was much higher than in devices with bare Au electrodes. More significant enhancements were found in *n*-channel characteristics. The linear *μ*_e_ of PTVPhI-Eh OFETs was increased by over 100 times by applying the PFO:s-SWNT and F8BT:s-SWNT interlayers with respect to the reference pristine devices. Interestingly, the linear mobilities show higher than saturation mobility, which indicates contact resistances were significantly reduced in the devices. In addition, the lower *V*_th_ and steeper *SS* originated from the enhanced charge injection. That is, the steeper *SS* originated from the reduction of capacitance at contact caused by the injection barrier between the metal electrode and the semiconductor^22^. The performance of the best devices was comparable to that of high-performance *n*-type polymer OFETs^37^. To check the generalizability of our interlayer functionality, we applied the interlayer to a commonly used *n*-type OSC, phenyl-C61-butyric acid methyl ester (PCBM), which showed similar enhancements in transistor characteristics (see Fig. S1 in Supplementary Information). This result indicates that our interlayers generally can improve charge injection in OFETs.

To check that the interlayers themselves do not act as conductive channels, we fabricated interlayer-only devices without an OSC layer. When F8BT:s-SWNT film (20 nm) of the same thickness as the OSC film was applied as a semiconducting layer in TGBC transistors, the devices showed extremely poor characteristics with 2.1 × 10^−4^ (for holes) and 3.4 × 10^−5^ cm^2^/V^−1^s^−1^ (for electrons) due to less dense percolation (see Fig. S2(a) and S2(b)) and the location of active channel. In the TGBC transistor, all charge carriers transport close to the semiconductor-dielectric interface. That is located at the top surface of the semiconductor film whereas s-SWNT network is located at the bottom of the semiconductor film. Moreover, the very low “OFF” current of PTVPhI-Eh OFETs seen in Fig. 2(a) confirms that there are no percolation paths through s-SWNTs in the interlayer matrix. Note that the increased “OFF” current with the interlayers often observed in the saturation regime is due to the more balanced ambipolarity caused by the increase of *n*-channel characteristics. In general, the ON/OFF ratios in ambipolar OFETs are significantly low and strongly dependent on *V*_ds_^4^,^38^. To explore the effects of metallic SWNTs on the interlayers, we dispersed SWNTs using poly(9,9-dioctylfluorene-co-bithiophene (F8T2), which is known to disperse both metallic SWNTs and s-SWNTs without selectivity^34^. Most of the devices with SWNT dispersed by F8T2 interlayer (F8T2:SWNT) exhibited metallic behaviors in the transfer characteristics due to the abundance of metallic SWNTs (Fig. S3(c)). Although one or two of nine devices showed enhanced characteristics with F8BT:s-SWNT and PFO:s-SWNT, the device stability in the cycling test was much lower than those OFETs with F8BT:s-SWNT (Fig. S4).

**Figure 3:**
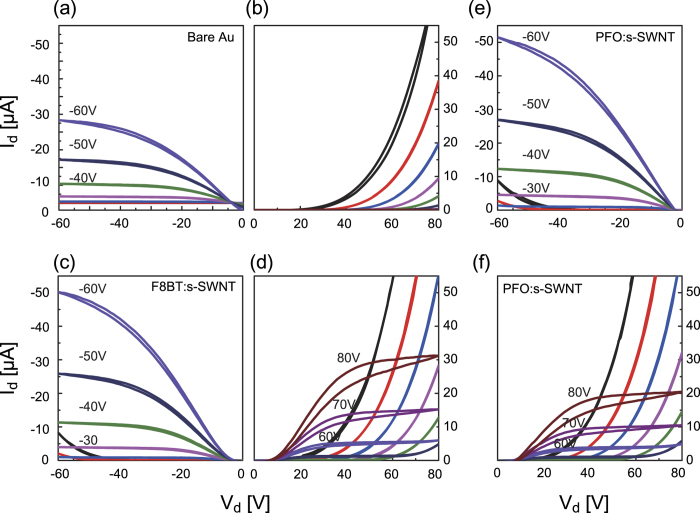
Output characteristics of PTVPhI-Eh OFETs with pristine gold electrodes (**a, b**), with F8BT:s-SWNT contacts (**c, d**), and with PFO:s-SWNT contacts (**e, f**).

Fig. 3 illustrates the output characteristics of ambipolar PTVPhI-Eh OFETs with Au or the interlayer coated on Au contact. The well-balanced ambipolar characteristics were observed from the output symmetry between *p*- and *n*-channel operations by applying the interlayer (Fig. 3c–f). When Fermi energy level of metal electrode was located in center position between HOMO and LUMO, the ambipolar injection become more balanced and efficient. Because of these reasons, it is difficult to be perfect Ohmic behavior in one polarity. Therefore, some output curves show the non-linear behavior. However, the current at small V_ds_ (i.e., in linear region) is considerably enhanced with respect to the pristine devices, indicating the much ameliorated charge injection and decreased R_c_.

**Figure 4:**
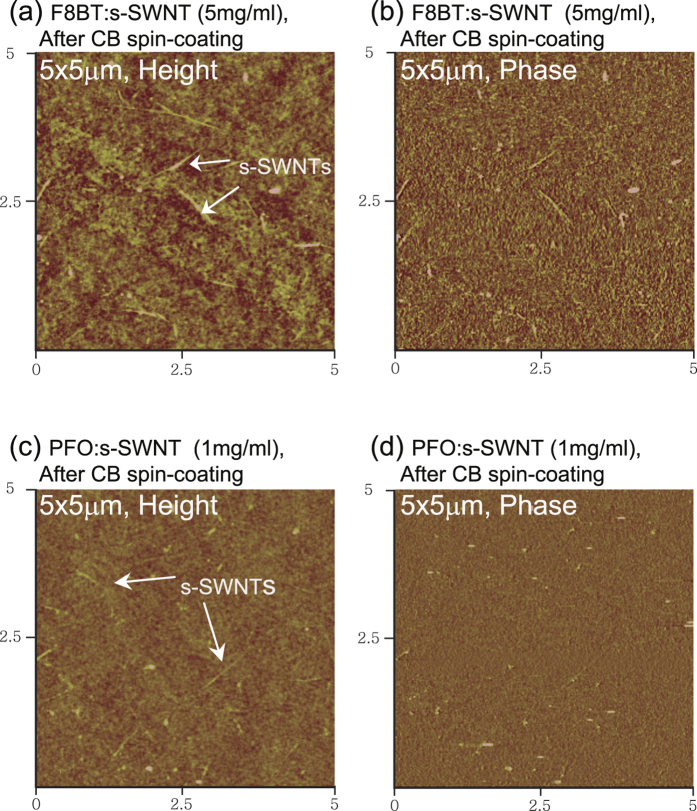
Tapping mode AFM images of F8BT:s-SWNT (**a, b**) and PFO:s-SWNT (**c, d**) thin films on Au after spin-coated chlorobenzene (CB) solvent (2000 rpm, 60 sec). Images on the left and right show topographic and phase mode images, respectively.

Fig. 4 shows the surface morphologies of the F8BT:s-SWNT and PFO:s-SWNT interlayers after the spin-coating of chlorobenzene (CB), which was used as the solvent of OSCs. After deposition of the interlayers onto Au followed by thermal annealing at 200 °C, CB was spin-coated on top of the interlayers. To check the wash off of F8BT and PFO in the interlayers during the deposition of the OSC, we compared the thicknesses of the interlayers before and after spin-coating of the CB solvent. After spin-coating of the CB solvent, the thicknesses of F8BT:s-SWNT and PFO:s-SWNT were slightly changed from 15 to 6 nm and 5 to 3 nm, respectively. Before washing by CB, the morphologies of the F8BT:s-SWNT and PFO:s-SWNT films showed several aggregated features, probably due to the SWNTs in the interlayer films (Fig. S5). In addition, we could not find any SWNT bundles at the sample surface before spin-coating of the CB solvent (Fig. S5), but SWNT bundles were distinguishably observed at the surface after spin-coating of the CB solvent (Fig. 4). The disclosure of SWNT at the surface is caused by the solubility difference between SWNTs and wrapping conjugated polymers to CB solvent. SWNTs produced by the HiPco process showed very low solubility to 1,2-dichlorobenzene and chloroform (95 and 31 mg·L^−1^) at room temperature, much lower than the solubility of PFO and F8BT (5–10 mg·mL^−1^)^8^,^39^,^40^,^41^. Thus, during the spin-coating of OSC, the F8BT and PFO on the s-SWNTs were naturally washed out at the interlayer surface, and the over-coated PTVPhI-Eh semiconducting film could obtain direct contact with s-SWNTs, which is crucial to improving both hole and electron injection. Furthermore, the morphological change of OSC between before and after deposition of interlayer did not observed (Fig. S5). The improvement of transistor characteristics is not due to morphological change of OSC.

**Figure 5:**
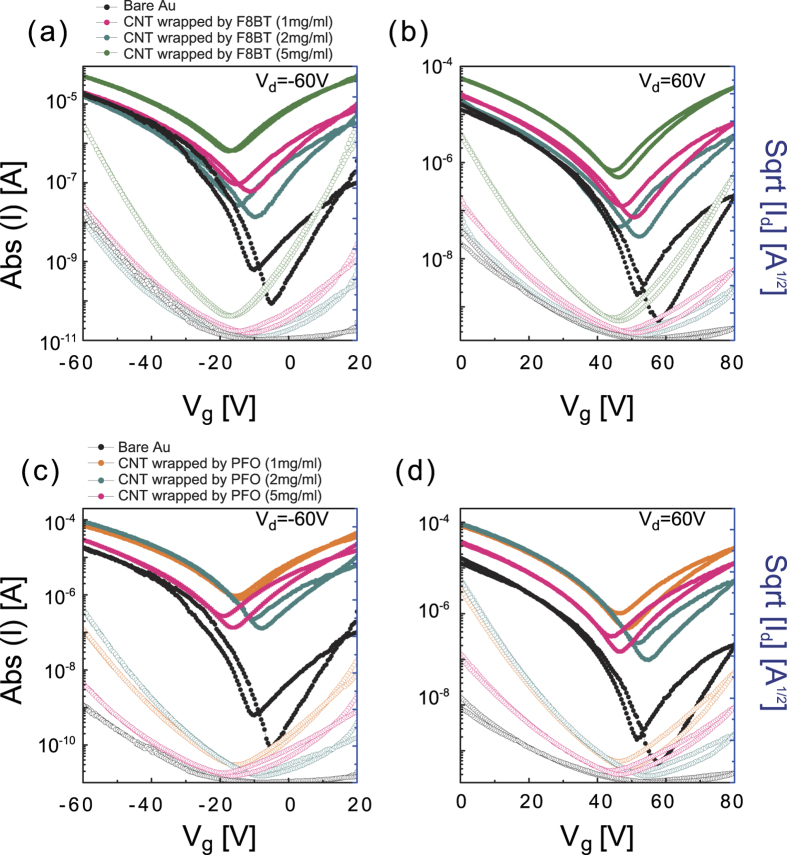
*P*-channel and *n*-channel transfer characteristics of OFETs with PFO:s-SWNT (**a, b**) and F8BT:s-SWNT (**c, d**) depending on SWNT wrapping polymer concentration.

To investigate the concentration effects of interlayer, F8BT or PFO solutions were prepared with various concentrations in toluene solvent for wrapping s-SWNT (Fig. S6). In this process as the concentration of the polymer solution increased, the number of wrapped s-SWNTs also increased (Fig. S6). However, higher concentrations of the polymer solution do not have much more s-SWNTs weight per polymer weight. In this article, s-SWNTs ratio in the hybrid solution is simply defined as below;
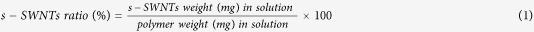


Fig. 5 shows the transfer curves of the OFETs containing s-SWNT interlayers obtained from polymer solution with various concentrations. The FET characteristics depend clearly on s-SWNTs ratio in the interlayer solution. More details are summarized in Table 2. When the PFO:s-SWNT solution were prepared with PFO of 1, 2, and 5 mg·ml^−1^, the s-SWNT ratio of PFO solution were 0.04, 0.02, and 0.03%, respectively. As the s-SWNT ratio increases in the solution compared to other solutions, the OFET performance is systematically improved. Similar results are observed in the OFETs incorporating F8BT:s-SWNT. For the most interlayers, the transistor’s contact properties are improved by increase of the thickness of the semiconductor interlayer up to a certain thickness and then it turns to degrade^20^. Unfortunately, in our system the exact interlayer thickness cannot be measured due to the washing off of interlayer. Instead of thickness, we suggest that “s-SWNT ratio” determines the property of charge injection. Interestingly, a very small number of s-SWNTs in the interlayer solution had a critical influence on OFET performance. For the F8BT:s-SWNT solution containing 1 and 2 mg·ml^−1^ F8BT, s-SWNT ratio were almost identical (0.006%). Despite the extremely low concentration of s-SWNTs, the OFETs with F8BT:s-SWNT, including 0.006% s-SWNT ratio, showed remarkably enhanced injection properties. The electron mobility was increased by approximately 3 times. This result supports the assertion that s-SWNTs play an important role, and even very small numbers of SWNTs significantly affect the charge injection of OFETs.

In order to verify the effects of wrapping polymers, we introduced the F8BT film (20 nm) only without s-SWNTs to the interlayer for PTVPhI-Eh OFETs (Fig. S7). The OFETs with the F8BT interlayer did not show any performance enhancement. Therefore, the observed improvement in the charge-injection properties of the OFETs was due mainly to the s-SWNTs rather than the wrapping of conjugated polymers. Furthermore, the very similar amount of improvement in the charge injection for OFETs with F8BT:s-SWNT and PFO:s-SWNT indicates that the chirality of s-SWNTs is not a dominant factor in charge injection, since PFO is capable of being selectively dispersed with five different sorts of s-SWNTs, whereas F8BT can wrap only two different s-SWNTs.

**Figure 6:**
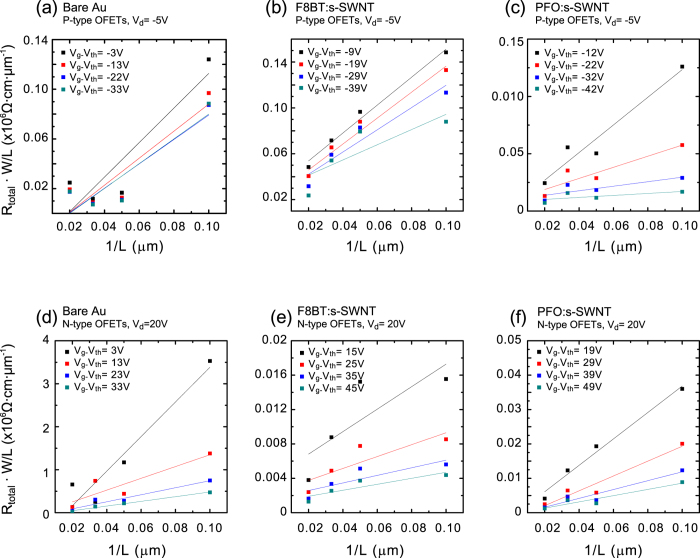
Modified transfer-line method plots for *p*- and *n*-channel PTVPhI-Eh OFETs with pristine gold electrodes (**a, d**), with F8BT:s-SWNT contacts (**b, e**), and with PFO:s-SWNT contacts (**c, f**). The slopes of the linear fit denote the *R*_c_.

The modified transfer-line method (M-TLM) was employed to determine *R*_*c*_ of the OFETs with diverse channel lengths (Fig. 6). The M-TLM allows a more reliable evaluation of *R*_*c*_ via a slight modification of the conventional TLM^42^. In the M-TLM, *R*_c_ is estimated from the slope of 1*/L* with *L* being the channel length, as described by the following equation^42^:
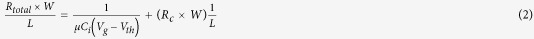
where *R*_total_ is the total resistance, *W* is the channel width, *C*_i_ is the unit area capacitance of the gate dielectric, and *V*_G_ is the gate voltage. Fig. 6 shows M-TLM plots of *p*- and *n*-channel PTVPhI-Eh OFETs with and without the interlayers. The extracted values of *R*_c_ from the pristine, F8BT:s-SWNT, and PFO:s-SWNT devices are 14, 9.6, and 1.9 MΩ·cm at V_g_ = −50 V in the *p*-channel regime and 400, 1.3, and 3.8 MΩ·cm at V_g_ = 50 V in the *n*-channel regime, respectively. The *R*_*c*_ was reduced by more than one order of magnitude for *p*-channel operation and by almost two orders of magnitude for *n*-channel operation. These results clearly indicate that charge injection is significantly improved by the insertion of the interlayers, particularly for electrons.

**Figure 7:**
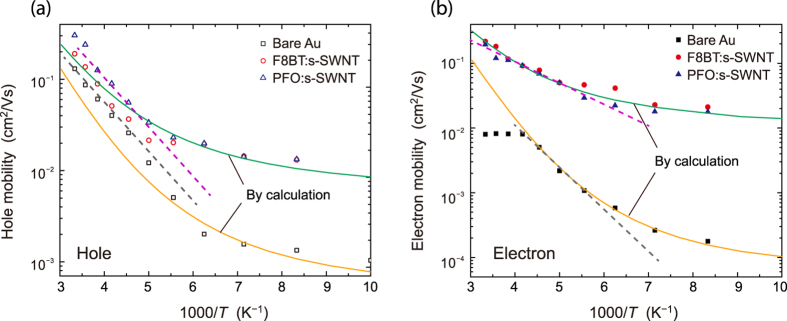
Temperature dependence of hole (**a**) and electron (**b**) mobilities and their calculation for OFETs with and without interlayers. Symbols represent experimental results, dashed lines at high temperatures are least-square fittings for extracting activation energy, and solid lines denote calculated data based on a previously reported mobility model.

In order to explore the underlying mechanism for the improved performance by using the interlayers, we performed a low-temperature measurement for all devices. The temperature-dependent hole and electron mobilities are shown in Fig. 7. In pristine PTVPhI-Eh OFETs, we extracted activation energies (*E*_a_) of 51 and 55 meV for hole and electron transport, respectively, by Arrhenius plotting. By incorporating the interlayers, the *E*_a_ declined to 50 and 27 meV for holes and electrons, respectively. The improved values of E_a_ for electron transport are comparable with those of state-of-the-art high-performance diketopyrrolopyrrole (DPP) derivatives^43^. Even the *E*_a_ for hole transport was not changed by the insertion of interlayers, yet the hole mobility was improved by nearly 10 times compared to that of pristine OFETs in the low-temperature regime, while this difference became smaller in the high-temperature regime. This is because at low temperatures, the macroscopic conductivity is primarily based on a single microscopic conductivity at Fermi energy so that the mobility is constant, and different charge concentrations (i.e., different Fermi levels) lead to different mobility magnitudes. At high temperatures, the larger number of microscopic conductivities spanning over a wide energy region concurrently contributes to the mobility, often manifesting as thermal activation. In addition, due to the presence of energetic disorder and the already-high Fermi level for hole transport (discussed below), the apparent *E*_a_ would not be very different before and after incorporating the interlayers^44^. Regarding the *E*_a_ for electron transport, more dramatic improvement was observed by inserting interlayers: Not only was the mobility magnitude improved over the entire temperature region, but also the activation energy was significantly reduced from 55 to 27 meV.

The decreased *E*_a_ for electron transport by adding the interlayers can be explained by the better electrical contacts between source/drain electrodes and the OSC. The *E*_a_ reflects the average barriers for the whole process of charge transport mainly via hopping from the source contact to the drain. Several factors could elevate the hopping barrier (e.g., traps at the contact region and in the channel that mainly result from contact properties, OSCs, the gate dielectric, and the OSC–dielectric interface)^45^,^46^. In this study the lowered *E*_a_ was due to the improved contact properties (i.e., reduced charge trapping at contacts) by the insertion of interlayers. It is logical that the enhanced charge injection supplies many more charge carriers to the channel. Thus, as the charge concentration is increased by adding interlayers, the Fermi level is accordingly raised, filling more gap states (traps) and freeing more injected carriers. This behavior is very similar to the abovementioned contact doping^47^. The increased density of mobile carriers by dopants raises the Fermi level and screens the lower energy states within the band gap of the OSC. On the other hand, when the interlayer is inserted, the π–π interaction and similar conjugated structure may reduce the trap states at metal–OSC interfaces^10^,^27^,^48^.

To comprehend how much the charge injection is improved more quantitatively and to understand why the mobility magnitude changes so dramatically by adding the interlayers, we implemented mobility calculations based on a solid mobility model that has been reported previously^44^,^47^. As there was only one difference in the contact interlayer, we kept the transport profile constant and only varied the charge concentration. Interestingly, the calculated data showed good agreement with our experimental results for both hole and electron mobility, as shown in Fig. 7. Here, the hole concentrations (*N*_h_) and electron concentrations (*N*_e_) of the pristine device and PTVPhI-Eh OFETs with the interlayers were 10^17.9^ and 10^18.5^ cm^−3^ and 10^16.7^ and 10^18.2^ cm^−3^, respectively. The data indicates that the application of the interlayers indeed increases the charge concentration owing to the improved charge injection. There are some features worthy of note. i) The original *N*_e_ = 10^16.7^ cm^−3^ is much lower than that of *N*_h_ = 10^17.9^ cm^−3^ due to the unbalanced charge injection in the pristine device. ii) The *N*_h_ is slightly increased to 10^18.5^ cm^−3^ by adding the interlayers, which explains the slight change in *E*_a_ for hole transport, since the Fermi level is already located at a high energy level. iii) Compared to *N*_h_, *N*_e_ is greatly enhanced to 10^18.2^, which is comparable to *N*_h_ in devices with the interlayers and is highly desirable for balanced ambipolar charge transport. The significantly increased electron density weakens the charge-trapping effects and lowers the hopping barriers, as is commonly observed in OFETs for gate-voltage enhanced mobility. Moreover, this improved charge injection for holes and electrons is accomplished by just one contact interlayer rather than the conventional interlayers specifically for either hole or electron injection. Therefore, truly high-efficient ambipolar transport can be expected in organic transistors.

We suggest two main mechanisms for effects of the interlayers on the improved hole and electron injection in OFETs. One is the strong 1D electrostatic effect of SWNTs, which have a form factor that associates with the 1D electrostatic field at the contact interfaces. The strong electric field induced by SWNTs at the contact region enables both electrons and holes to tunnel from metal electrodes to OSCs. Some research groups have reported such 1D electrostatic effects on the SWNT-included electrode and the relevant transistors experimentally and theoretically.^49-52^ In this context, the 1D electrostatic field significantly reduced the depletion thickness to a few nanometers, facilitating efficient charge injection by tunneling^8^,^26^,^49^,^50^,^51^,^52^. Second mechanism is the doping effect of the active layer in OFETs by SWNTs via the charge transfer between the conjugated polymer and SWNT^53^,^54^,^55^,^56^. Silva *et al.* reported an electron transfer from SWNT to P3HT, resulting to P3HT and SWNT were n-doped and p-doped respectively^56^. We conjecture that more highly enhanced *n*-type characteristics than *p*-type may be originated from combination of the doping and electrostatic effect. Here, although the s-SWNT interlayer was used with conjugated polymers, a similar injection principle would also be applicable. The energetic levels of the interlayers were checked by ultraviolet photoemission spectroscopy (UPS), but the data did not match our device characteristics (Fig. S8).

**Figure 8:**
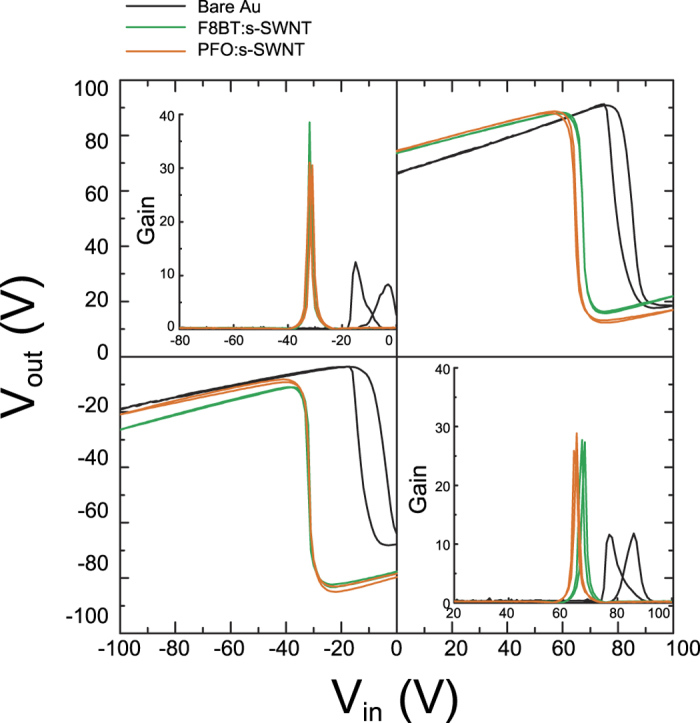
Voltage transfer characteristics of ambipolar complementary inverters based on PTVPhI-Eh OFETs [*W*/*L* ratios for *p*- and *n*-channel, *W*/*L = *1 mm/20 μm]: bare Au S/D electrode (black); and, with F8BT:s-SWNT (green) and PFO:s-SWNT interlayer (yellow).

Finally, we demonstrated ambipolar complementary inverters that were fabricated via a spin-coating process without sophisticated patterning for *p*- and *n*-channel regions (Fig. 8). Each inverter comprised two identical ambipolar transistors. Fig. 8 shows the voltage transfer characteristics (VTCs) of PTVPhI-Eh inverters with and without the interlayers. As the unique characteristics of ambipolar inverter, the PTVPhI-Eh inverter operated not only positive but also negative input voltage (V_in_) depending on the polarity of the supply voltage, which cannot be operated in unipolar inverter^57^,^58^. In conventional complementary inverter which is consist of *p*-type and *n*-type unipolar transistors, V_in_ alternatively switches on and off the *p*-and *n*-type transistors, respectively, (i.e., the pull-up and pull-down transistors). Whereas in an ambipolar complementary inverter, pull-up and pull-down transistor are consist of the same ambipolar transistor. Therefore, our inverter worked both positive and negative V_in_. The inverters with the interlayers manifested much better switching characteristics at the input *V*_in_ very close to half the power supply voltage (*V*_dd_/2). Furthermore, the significantly improved voltage gains of 40 and 30 from 10 for the pristine device were achieved by incorporation of the F8BT:s-SWNT and PFO:s-SWNT interlayers, respectively. These results rely on the balanced *μ*_h_ and *μ*_e_ and matched *V*_th_ in the *p*- and *n*-channel regimes, thanks to the improved ambipolar charge injection.

## Conclusion

In summary, we achieved charge-injection improvement for both holes and electrons in ambipolar OFETs by using interlayers fabricated by conjugated polymer -wrapping s-SWNTs with a low-speed centrifugation. Thus, no additional process was needed to remove excess conjugated polymers. The kinds of conjugated polymers and the chirality of s-SWNTs were not important factors in improved OFETs with the interlayers. In case of interlayer with abundant metallic SWNT, the operational stability was even lower than those OFETs with interlayers containing s-SWNTs. s-SWNTs themselves played an important role, and even a very small number of s-SWNTs significantly affected the charge injection of OFETs. We concluded that interlayers provide much better interfacial contact than the Au–OSC interface and increase the charge concentration. The improved injection of both holes and electrons was qualitatively considered by the tunneling across the Schottky barrier because of the 1D electrostatic effects caused by the form factor of the SWNTs. Finally, we demonstrated a well-balanced ambipolar CMOS-like inverter with the interlayers, which achieved the high gain of 40. We believe that these interlayers prepared with this simple method are not only effective for ambipolar charge injection in most organic optoelectronic devices with contact problems, but also highly compatible with solution-processed manufacturing processes without micropatterning for fully printed organic optoelectronic devices.

## Method

### Preparation of Conjugated Polymer-wrapping SWNT Interlayer Solution

The polymers used in this study were PFO (Sigma Aldrich, M_w_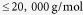) and F8BT (American Dye Source Inc., M_w_ ≈ 15,000 – 200,000). SWNTs grown by the HiPco process (diameter 0.8–1.2 nm, purified <13 wt% iron) were purchased from Unidym Inc. All other chemicals were purchased from Sigma Aldrich. The toluene solution of PFO (1, 2, and 5 mg/ml) and F8BT solution (1, 2, and 5 mg/ml) were prepared and heated at 80 °C for 2 hr for complete dissolution. After cooling, the SWNT powder was added to each solution at 0.5, 1, and 2.5 mg/ml, and the solutions were homogenized in an ultrasonic bath (Branson 5510) for 30 min, followed by probe tip sonication (VCX 750 Ultrasonic Processor with Temperature Probe) for 15 min. Then, these resulting solutions were centrifuged (VS-15000CFNII, Vision Scientific), and the supernatants were collected.

## Fabrication of OFETs

Interdigitated source/drain electrodes on Corning Eagle 2000 glass substrates were processed in sequence by photolithography, thermal evaporation of 5 nm-thick nickel and 15 nm-thick gold, and lift-off (with channel width *W* = 1 mm, channel length *L* = 20 μm). The substrates were cleaned in an ultrasonic bath with deionized water, acetone, and 2-propanol for 10 min each. The substrates and electrodes were treated with UV/ozone for 20 min before the deposition of interlayers. The polymer/SWNT dispersion solution was spin-coated onto substrates at 2000 rpm for 60 s, and the resulting films were annealed in air at 200 °C for 20 min. An ambipolar semiconductor PTVPhI-Eh (M_w_ = 35,800 g/mol) was synthesized for this work, as reported elsewhere^59^. PTVPhI-Eh and PC_60_BM were dissolved in anhydrous CB to obtain 5 mg/ml and 10 mg/ml concentrations, respectively, and then they were spin-coated at 2000 rpm in a dry nitrogen glovebox. The semiconducting films were annealed at 110 °C for 20 min on a hotplate to remove residual solvent and moisture, and the film thickness measured by an atomic force microscope (AFM) was uniformly ~20 nm. For the dielectric of PTVPhI-Eh OFETs, poly(methyl methacrylate) (PMMA) (M_w_ = 120kD) was used without further purification and dissolved in n-butyl acetate (80 mg/ml). The dielectric solution was spin-coated on top of the semiconducting film at 2000 rpm for 60 s, and the final thickness was approximately 500 nm. For the dielectric of PC_60_BM OFETs, the fluoropolymer CYTOP (Asahi Glass Co.) was chosen, because the PC_60_BM film is a small molecular OSC that can be dissolved by the nBA of PMMA solvent. Spin-coating CYTOP (CYTOP: solvent = 2:1) on top of the PC_60_BM formed the dielectric layer (approximately 350 nm determined by a surface profiler (Surf Cored ET 3000)). The devices were baked at 80 °C for 30 min under nitrogen conditions. The aluminum top-gate electrodes (thickness of ~50 nm) were thermally evaporated through shadow masks in a high vacuum chamber (~10^6^ Torr). Reference devices without polymer/SWNT interlayers were processed in the same way.

## Characterizations

UV-Vis spectra were measured using a Perkin-Elmer Lambda 750 instrument. The FET electrical characterizations were carried out using a semiconductor parameter analyzer (Keithley 4200-SCS) in an N_2_-filled glovebox. Linear charge carrier mobility values were extracted by the M-TLM plots^42^, following the expression (1). In the equation, the V_th_s of each devices were extrapolated from linear segment of transfer curve. Saturation charge carrier mobility values were extracted by the transfer curves according to the expression 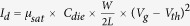, where Id is the drain current, 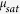
is the saturation mobility. The height and phase images of the F8BT:s-SWNT and PFO:s-SWNT were obtained using an AFM (Nanoscope III, Veeco Instruments, Inc.) at the Korea Basic Science Institute (KBSI).

## Mobility Calculations

The mobility calculations were performed using numerical software with a previously reported mobility model. It is based on the Kubo–Greenwood integral developed by Sir Nevill Francis Mott and coworkers, which described the relationship between mobility and transport properties in non-crystalline semiconductors, including amorphous silicon conductors and OSCs. As the contact interlayers did not affect the transport profiles of holes and electrons in the channel, the relevant parameters were kept identical in calculating the mobility for the OFETs with and without interlayers, and only one factor was changed: the charge concentration. The densities of states in the HOMO and LUMO of PTVPhI-Eh were supposed to be 10^21^ cm^−3^ with a Gaussian distribution in energy, and their standard deviation ∆*E* reflects the energetic disorder. Another parameter of the applied mobility model is *α*, which is a hybrid level of delocalized and localized states in the band. A maximum value of *α* = 1 indicates fully band-like transport, while a smaller *α* indicates a greater hopping contribution to the overall transport process. In this study, ∆*E* = 0.12 eV and *α* = 0.56 for hole transport, and ∆*E* = 0.12 eV and *α* = 0.6 for electron transport. The HOMO and LUMO levels of PTVPhI-Eh were set to be 5.24 eV and 3.39 eV lower than the vacuum level, respectively, and the relative permittivity of PTVPhI-Eh was configured as 2.1.

## Additional Information

**How to cite this article**: Lee, S.-H. *et al.* Simultaneous Improvement of Hole and Electron Injection in Organic Field-effect Transistors by Conjugated Polymer-wrapped Carbon Nanotube Interlayers. *Sci. Rep.* **5**, 10407; doi: 10.1038/srep10407 (2015).

## Supplementary Material

Supporting Information

## Figures and Tables

**Table 1:** Fundamental parameters of TGBC OFETs based on PTVPhI-Eh and after deposition of F8BT:s-SWNT and PFO:s-SWNT onto Au S/D electrode*.

	**P-channel**					**N-channel**				
**Interlayer**	**μ**_**h**_**(cm**^**2**^**V**^**-1**^**S**^**-1**^)			**V**_**th**_	**SS (V/dec.)**	**μ**_**e**_**(cm**^**2**^**V**^**-1**^**S**^**-1**^)			**V**_**th**_	**SS V/dec.)**
	**Saturation**		**Linear**			**Saturation**		**Linear**		
	**Aver.**	**Max.**				**Aver.**	**Max.**			
Without interlayer	0.11 (±0.01)	0.13	0.05	−45.62 (±5.3)	2.6 (±0.28)	0.001 (±0.0004)	0.002	6.5×10^−3^	47.2 (±2.2)	4.98 (±1.13)
F8BT:s-SWNT (5mg/ml)	0.24 (±0.05)	0.3	0.06	−21.65 (±1.01)	0.94 (±0.32)	0.16 (±0.03)	0.22	0.26	35.28 (±1.59)	2.88 (0.59)
PFO:s-SWNT (1mg/ml)	0.34 (±0.03)	0.38	0.5	−18.73 (±1.25)	0.93 (±0.3)	0.11 (±0.03)	0.16	0.53	31.28 (±2.08)	2.6 (±0.56)

*μ_h_ and *μ*_e_ refer to the field-effect mobility measured in the linear (*V*_ds_ = −5 V for holes, and *V*_ds_ = 20 V for electrons) and saturation (*V*_ds_ = ± 60 V for holes and electrons) regimes. The threshold voltages (V_th_) and subthreshold swing (*SS*) were extracted from the linear regime. The OFET saturation mobilities extracted from standard FET I-V equation (see experimental section) and listed in this table are averages of at least five devices (*L* = 20 μm and *W* = 1 mm), and the error bars denote the standard deviations. The OFET linear mobilities extracted at from M-TLM equation which in contact resistances are reflected. The V_g_-V_th_ in p-channel of bare, F8BT:s-SWNT and PFO:s-SWNT are each −13, −9 and −12 V. The V_g_-V_th_ in n-channel of bare, F8BT:s-SWNT and PFO:s-SWNT are each 13, 15 and 19 V.

**Table 2:** Hole and electron saturation mobilities of PTVPhI-Eh OFET with or without PFO:s-SWNT and F8BT:s-SWNT interlayers at various concentrations.

**Interlayers**	**Concentration of solution**	^**a**^**SWNT concentration (μg/ml)**	**s-SWNTs ratio (%)**	^**b**^***μ***_**h**_**(cm**^**2**^**V**^**−1**^**S**^**−1**^**) Average**	^**b**^***μ***_**e**_**(cm**^**2**^**V**^**−1**^**S**^**−1**^**) Average**
Without interlayer	-	-	-	0.11 (±0.01)	0.001 (±0.0004)
	PFO solution 1 mg/ml	~0.4	0.04	0.343 (±0.034)	0.11 (±0.03)
PFO:s-SWNT	PFO solution 2 mg/ml	~0.5	0.02	0.361 (±0.19)	0.034 (±0.01)
	PFO solution 5 mg/ml	~1.5	0.03	0.21 (±0.025)	0.09 (±0.011)
	F8BT solution 1 mg/ml	~0.06	0.006	0.13 (±0.045)	0.046 (±0.045)
F8BT:s-SWNT	F8BT solution 2 mg/ml	~0.12	0.006	0.09 (±0.021)	0.03 (±0.008)
	F8BT solution 5 mg/ml	~1.2	0.026	0.24 (±0.03)	0.16 (±0.03)

^a^Calculated from UV-Vis-NIR spectrum according to absorption cross-section of 1.7 × 10^−17^cm^2^ for E^11^ absorption peak^36^.

^b^Calculated from curve of *I*_ds_^1/2^ versus *V*_g_ in saturation regime (at *V*_d_ = ± 60).
