# Supplementary material for: Simultaneous Improvement of Hole and Electron Injection in Organic Field-effect Transistors by Conjugated Polymer-wrapped Carbon Nanotube Interlayers
Source: Sci Rep. 2015 May 22;5:10407. doi: 10.1038/srep10407 (PMC5377053; doi:10.1038/srep10407)
Supplement: Supporting Information [file srep10407-s1.docx]

Supplementary Information

Simultaneous Improvement of Hole and Electron Injection in Organic Field-effect Transistors by Conjugated Polymer-wrapped Carbon Nanotube Interlayers

*Seung-Hoon Lee, Dongyoon Khim, Yong Xu, Juhwan Kim, Won-Tae Park, Dong-Yu Kim*, and Yong-Young Noh**


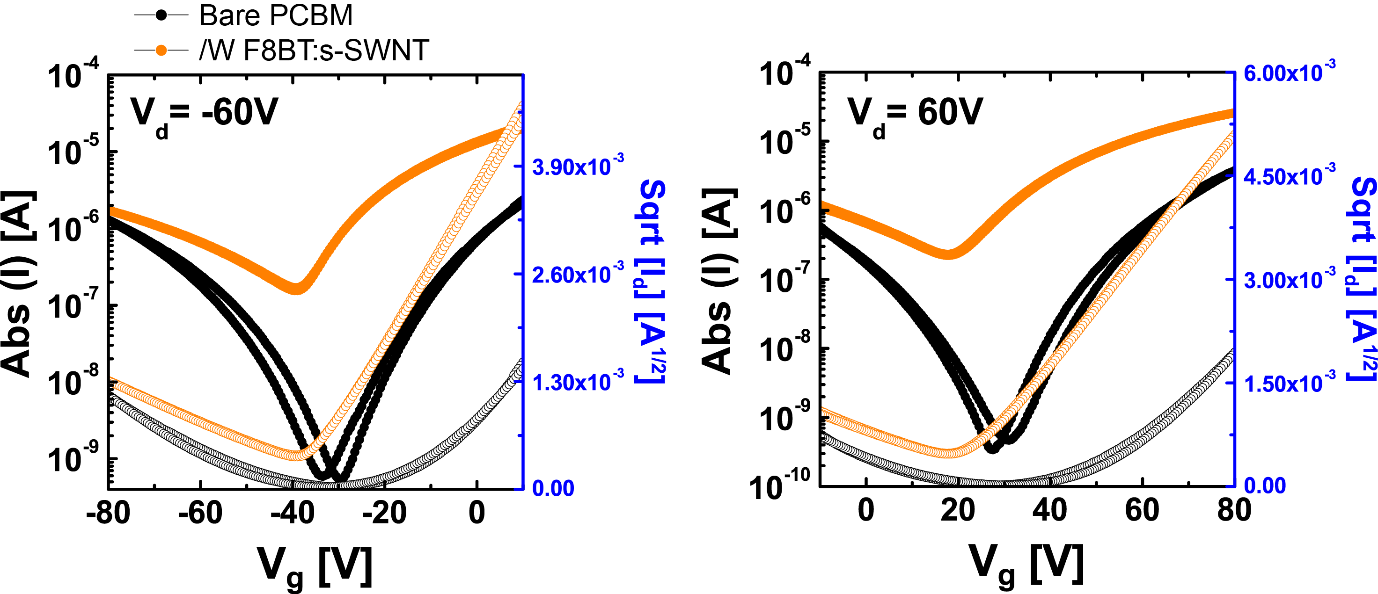


Figure S1. P-channel and n-channel transfer characteristics of ambipolar PCBM OFETs using s-SWNTs wrapped by F8BT interlayers (*L*=20 μm, *W*/*L*=50, *C*_i_=6.2 nFCm^−2^).


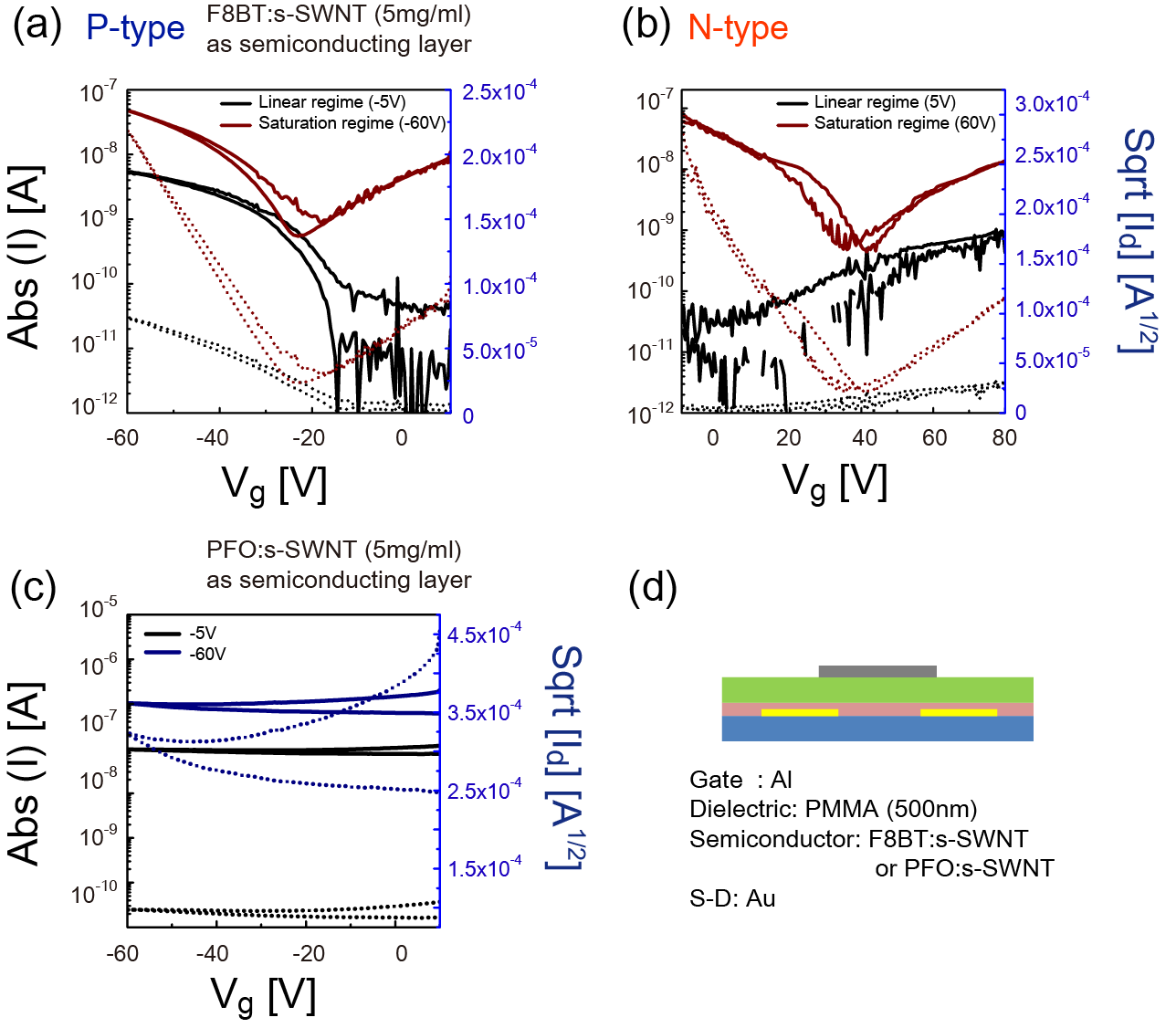


Figure S2. P-channel and n-channel transfer characteristics of OFETs with F8BT:s-SWNT film (a,b) or PFO:s-SWNT (c) as a semiconducting layer (*L*=20 μm, *W*/*L*=50, *C*_i_=6.2 nFCm^−2^). (d) Schematic of OFET structure of interlayer-only device.


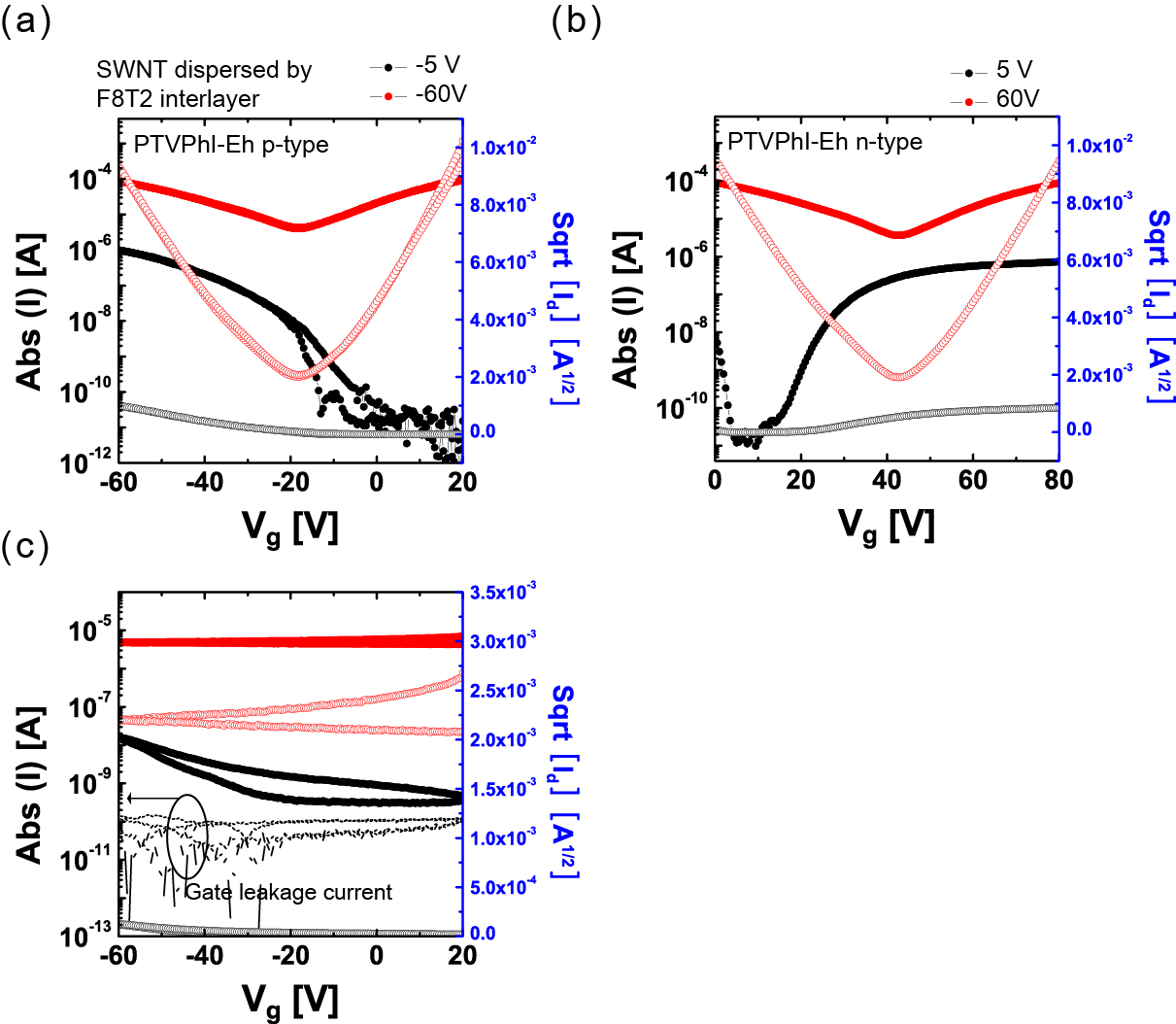


Figure S3. P-channel (a) and n-channel, (b) transfer characteristics of PTVPhI-Eh OFETs using SWNT dispersed by F8T2 interlayers (a few devices), (c) Metallic behaviors in transfer characteristics due to abundant metallic SWNT in most devices with SWNT dispersed by F8T2 interlayers (*L*=20 μm, *W*/*L*=50, *C*_i_=6.2 nFCm^−2^).


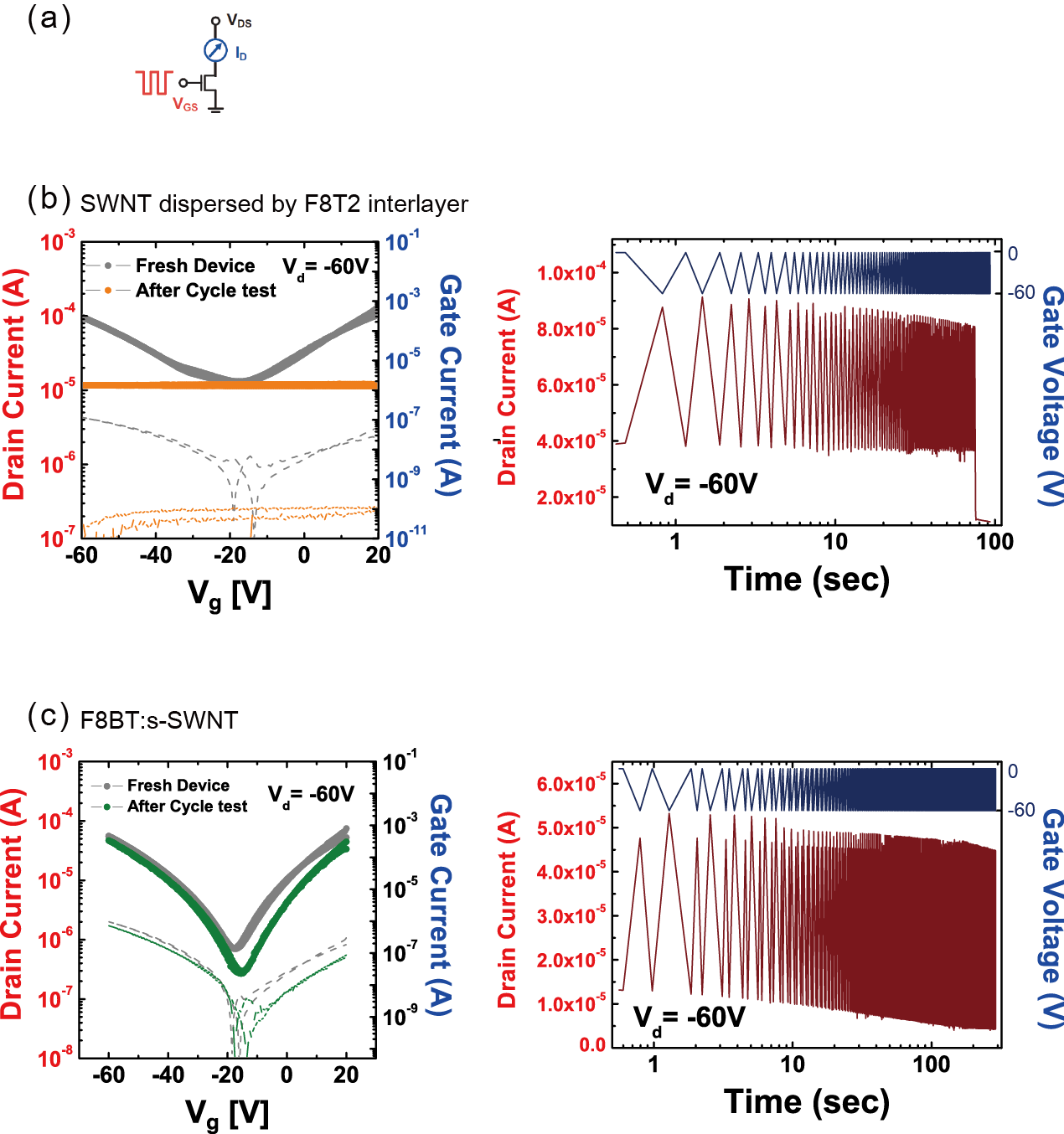


Figure S4. (a) Schematic of cycle test, (b) Electrical characteristics of PTVPhI-Eh OFET with SWNT dispersed by F8T2 interlayer fresh and after cycle test, (c) Electrical characteristics of PTVPhI-Eh OFET with F8BT:SWNT fresh and after cycle test (*L*=20 μm, *W*/*L*=50, *C*_i_=6.2 nFCm^−2^).


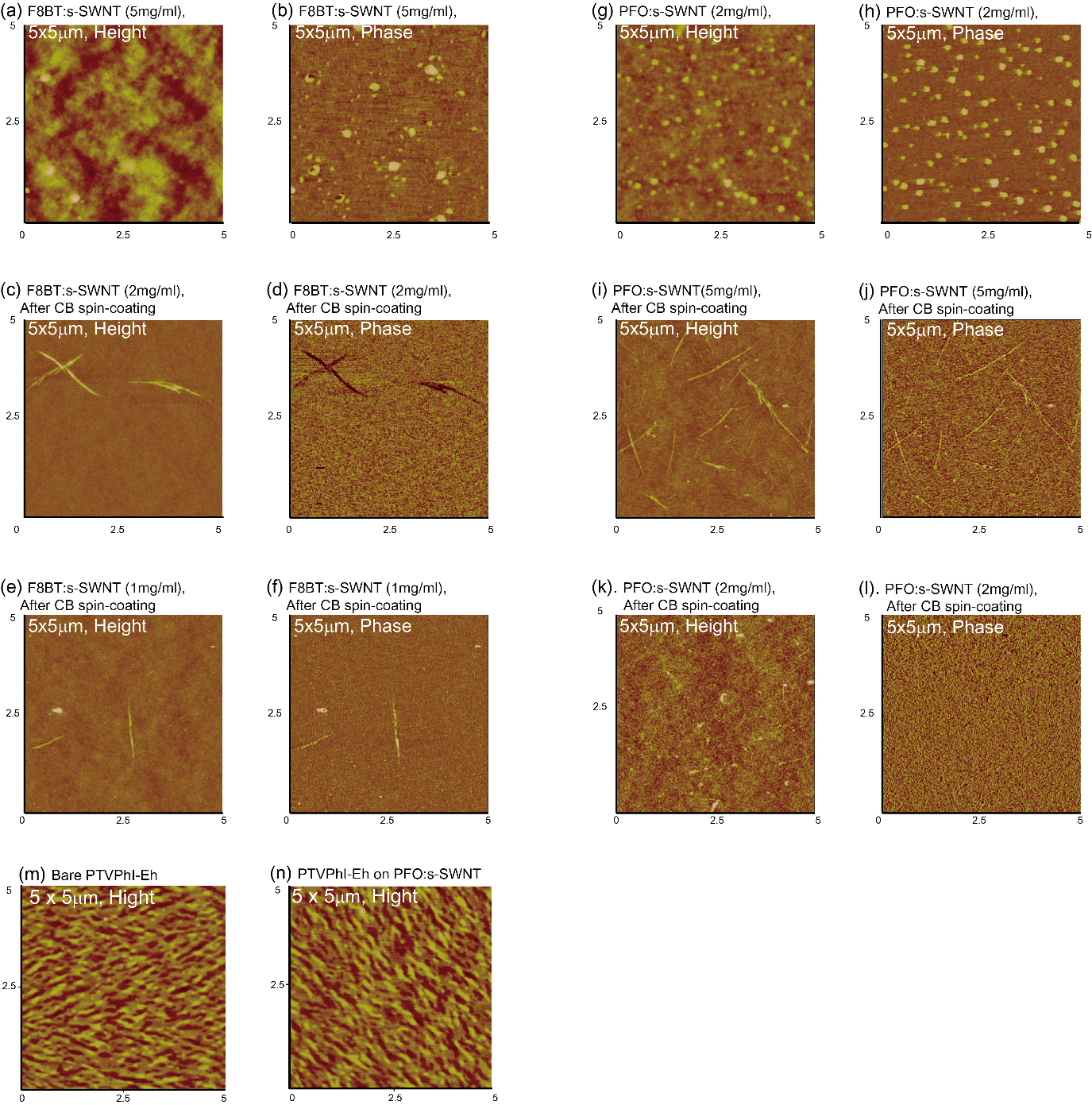


Figure S5. Tapping mode AFM images. (a) Height image, (b) Phase image of F8BT:s-SWNT (5mg·ml^-1^), (c) Height image, (d) Phase image of F8BT:s-SWNT (2mg·ml^-1^) after CB spin-coating, (e) Height image, (f) Phase image of F8BT:s-SWNT (1mg·ml^-1^) after CB spin-coating, (g) Height image, (h) Phase image of PFO:s-SWNT (2mg·ml^-1^), (i) Height image, (j) Phase image of PFO:s-SWNT (5mg·ml^-1^), (k) Height image, and (l) Phase image of PFO:s-SWNT (2mg·ml^-1^) after CB spin-coating on Au surfaces. (m) bare OSC and (n) on PFO:s-SWNT


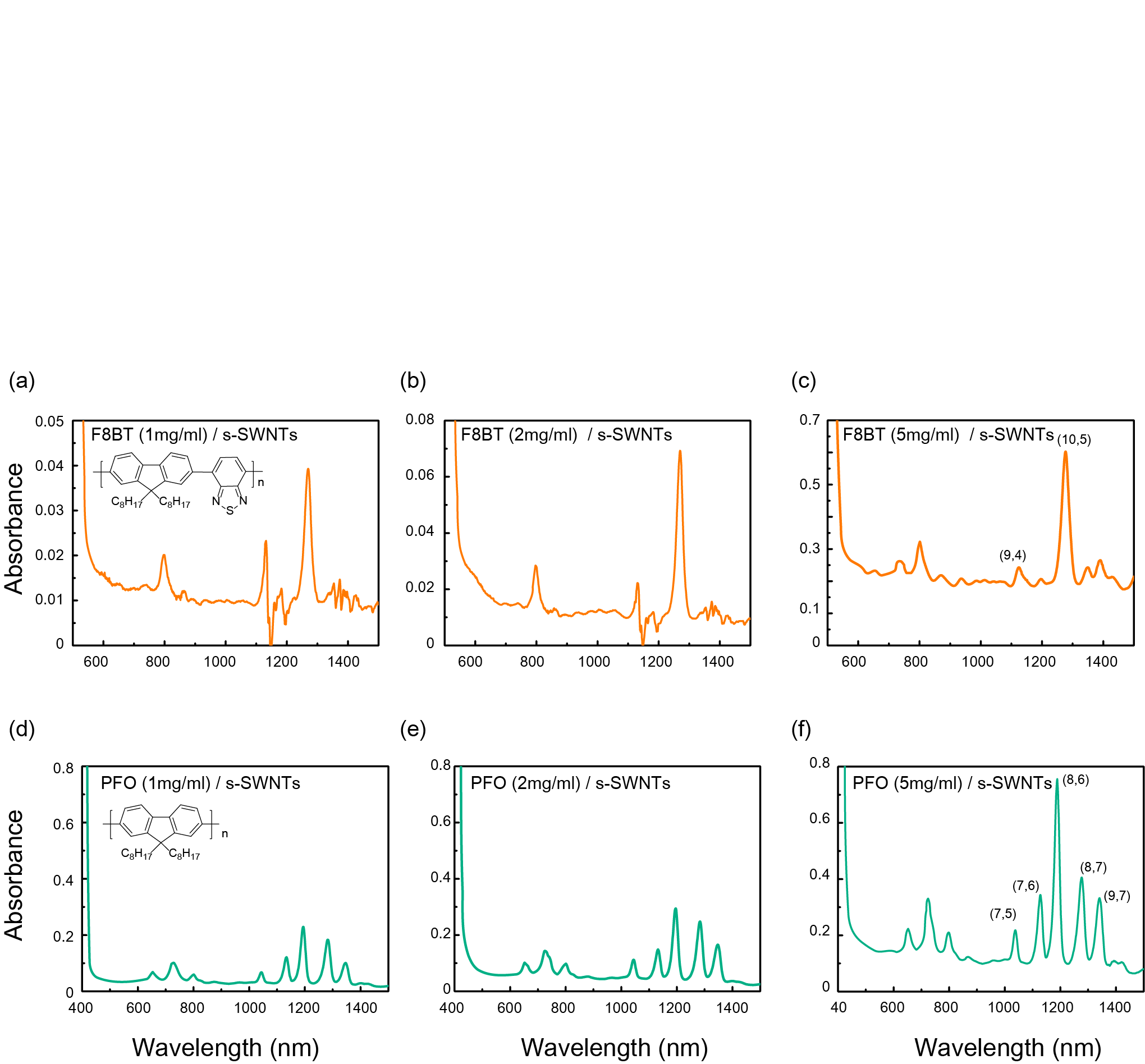


Figure S6. UV/Vis-NIR absorption spectra of F8BT:s-SWNT (a, b, and c) and PFO:s-SWNT (d, e, and f) in toluene solution with different polymer concentrations.


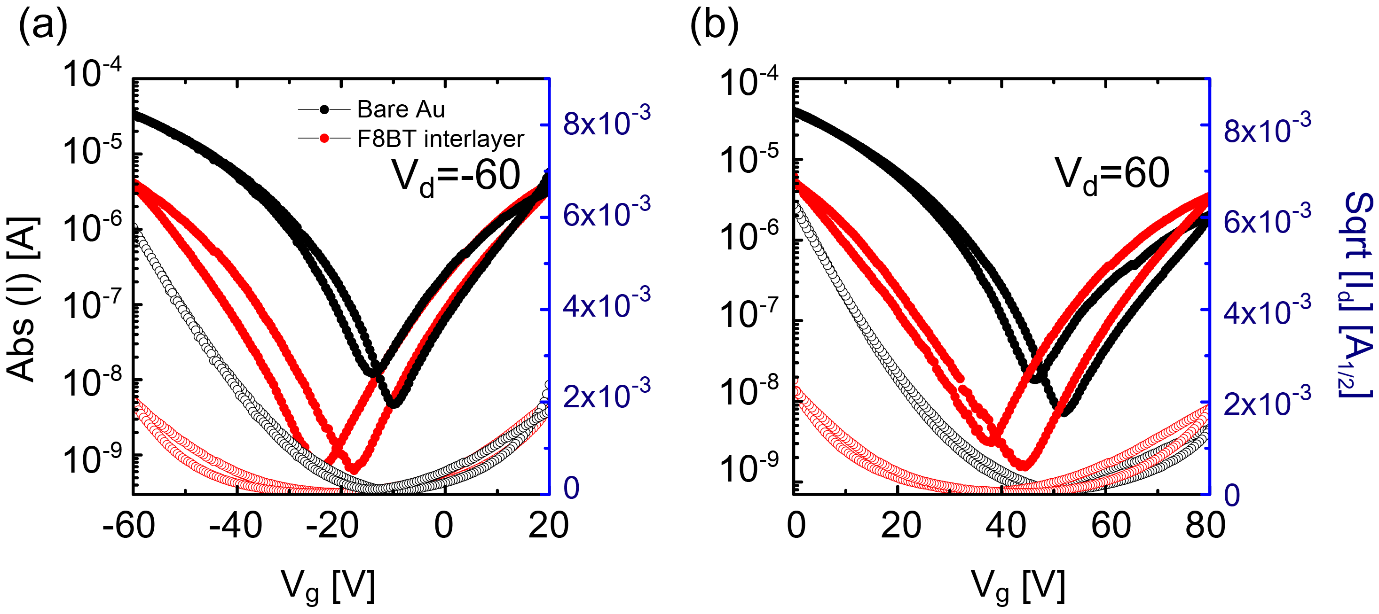


Figure S7. P-channel and n-channel transfer characteristics of ambipolar PTVPhI-Eh OFETs using only F8BT interlayers without SWNT (*L*=20 μm, *W*/*L*=50, *C*_i_=6.2 nFCm^−2^).


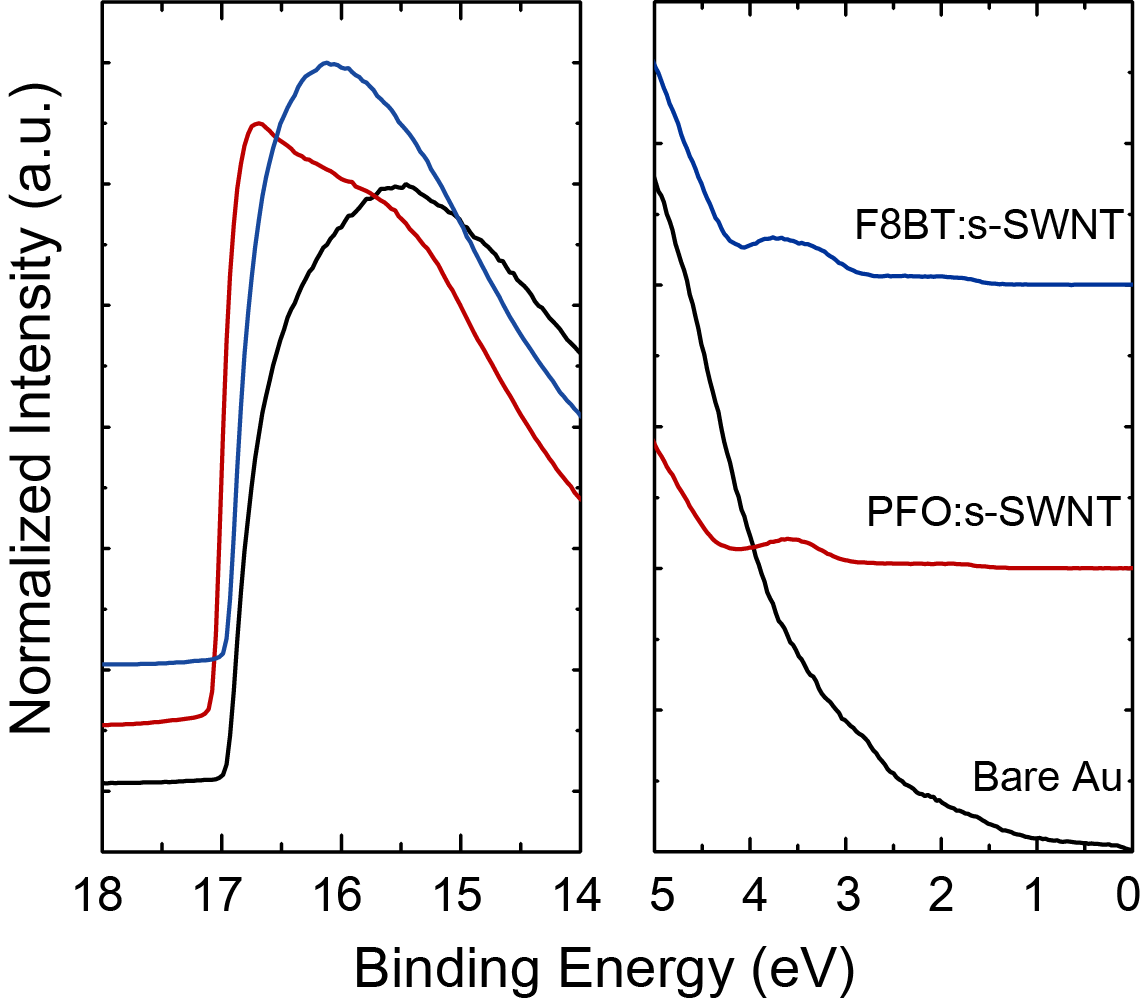


Figure S8. UPS spectra of various interfacial layers on Au. HOMOs of PFO:s-SWNT and F8BT:SWNT are 5.58 and 5.73 eV, respectively.


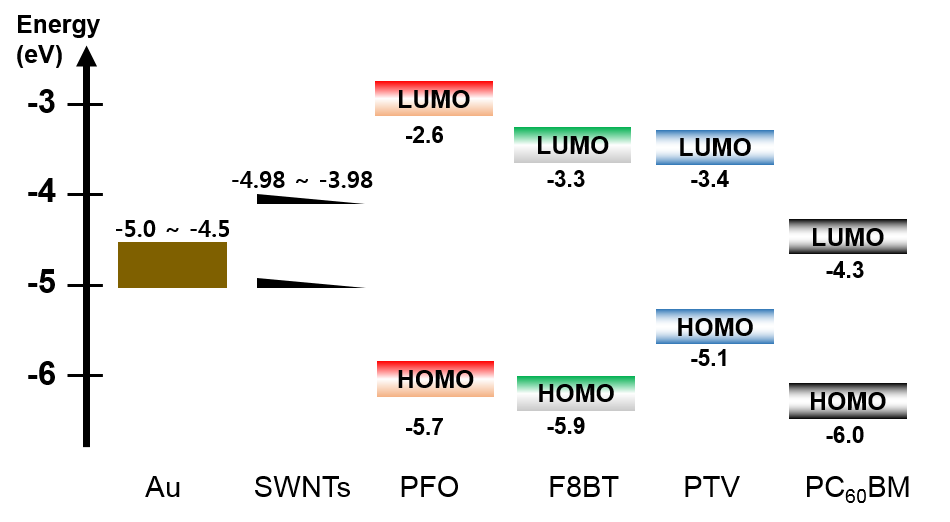


Figure S9. Energy diagram of s-SWNTs according to Tanaka et al.,^1^ wrapping polymers and PTVPhI-Eh

Table S1. HOMOs, LUMOs, length and nanotube diameter of SWNT in Hipco sorted by PFO and F8BT.^1^

|  | Chirality | Nanotube diameter [nm] | HOMO | LUMO |
| --- | --- | --- | --- | --- |
| SWNT sorted by PFO | (7,5) | 0.829 | 4.98 | 3.97 |
|  | (7,6) | 0.895 | 4.94 | 4.03 |
|  | (8,6) | 0.966 | 4.9 | 4.05 |
|  | (8,7) | 1.032 | 4.88 | 4.09 |
|  | (9,7) | 1.103 | 4.85 | 4.1 |
| SWNT sorted by F8BT | (9,4) | 0.916 | 4.92 | 4.01 |
|  | (10,5) | 1.050 | 4.86 | 4.08 |

**Reference**

1. Yasuhiko T. et al, Experimentally Determined Redox Potentials of Individual (n, m) Single‐Walled Carbon Nanotubes. *Angew. Chem., Int. Ed.* 48, 7655 (2009)

End
